# Supplementary material for: Initial cell density encodes proliferative potential in cancer cell populations
Source: Sci Rep. 2021 Mar 17;11:6101. doi: 10.1038/s41598-021-85406-z (PMC7969775; doi:10.1038/s41598-021-85406-z)
Supplement: Supplementary file 1 — Supplementary information. [file 41598_2021_85406_MOESM1_ESM.pdf]

Initial cell density encodes proliferative potential in cancer cell populations - Supplementary Information

Chiara Enrico Bena[1,2], Marco Del Giudice[2,3], Alice Grob[4,9], Thomas Gueudré[2], Mattia Miotto[5], Dimitra Gialama[6], Matteo Osella[7], Emilia Turco[8], Francesca Ceroni[6,9], Andrea De Martino[10,2], and Carla Bosia[11,2,*]

[1]Sorbonne Université, CNRS, Institut de Biologie Paris-Seine (IBPS), Laboratoire Jean Perrin (LJP), F-75005, Paris, France
[2]IIGM - Italian Institute for Genomic Medicine, c/o IRCCS, Str. Prov.le 142, km 3.95, 10060, Candiolo, Italy
[3]Candiolo Cancer Institute, FPO-IRCCS, Str. Prov.le 142, km 3.95, 10060, Candiolo, Italy
[4]Department of Life Sciences, Imperial College London, London, UK
[5]Department of Physics, Sapienza University, Piazzale Aldo Moro 5, 00185, Rome, Italy
[6]Department of Chemical Engineering, Imperial College London, London, UK
[7]Physics Department and INFN, University of Turin, Via P. Giuria 1, 10125, Turin, Italy
[8]Molecular Biotechnology Center, University of Turin, Via Nizza 52, 10126, Turin, Italy
[9]Imperial College Centre for Synthetic Biology, London, UK
[10]Soft & Living Matter Lab, CNR-NANOTEC, Rome, Italy
[11]Department of Applied Science and Technology, Politecnico di Torino, Corso Duca degli Abruzzi 24, 10129, Turin, Italy
[*]Corresponding author: carla.bosia@polito.it

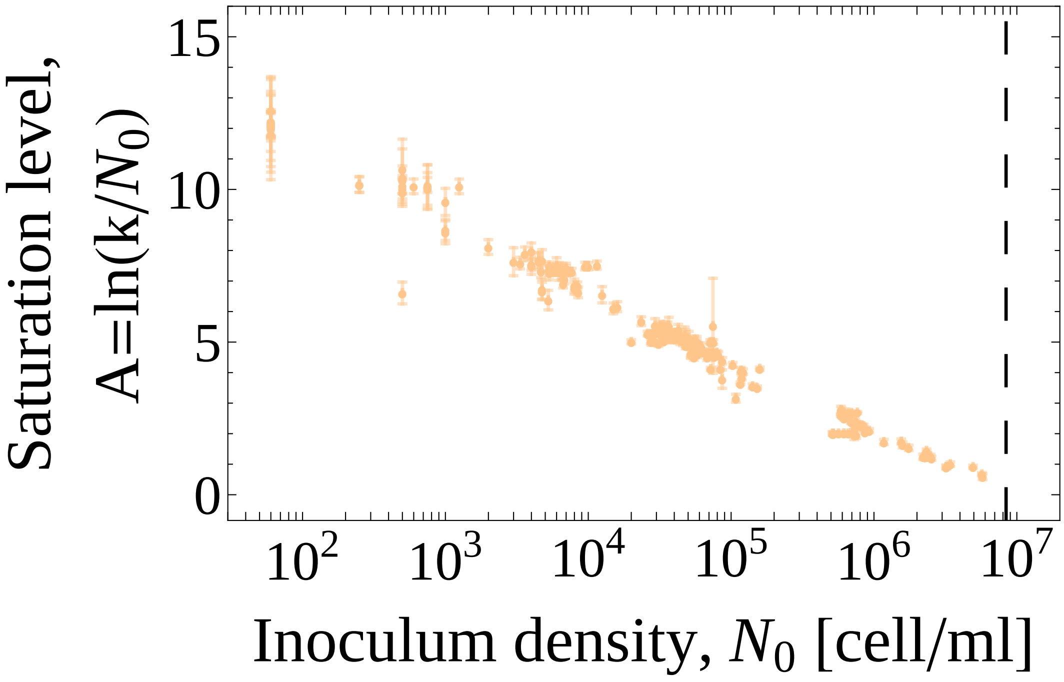

Figure S1. The saturation level A of the growth curves (Jurkat cells) is plotted as a function of the inoculum density N_0. Each dot corresponds to a specific experiment and carries its own standard error.

Data robustness with respect to the fitting function

The modified logistic function used in the main text to fit growth curves did not have any modeling purpose and has been used as a mere tool to quantify the macroscopic variables that characterize growth. To test the robustness of our results, we verified their independence on the fitting function. For such purpose, we fitted the same growth curves with a modified Gompertz function[1] whose parameters are the same of the modified logistic function (maximum growth rate λ_{max}, lag time t_{lag} and growth saturation level A):

$$\ln(N/N_0) = A\exp\left\{-\exp\left[\frac{e\lambda_{max}}{A}(t_{lag}-t)+1\right]\right\} \quad . \tag{1}$$

Figure S2 shows that results obtained by the two fitting procedures are compatible. Our scenario is therefore robust to the use of different fitting functions.

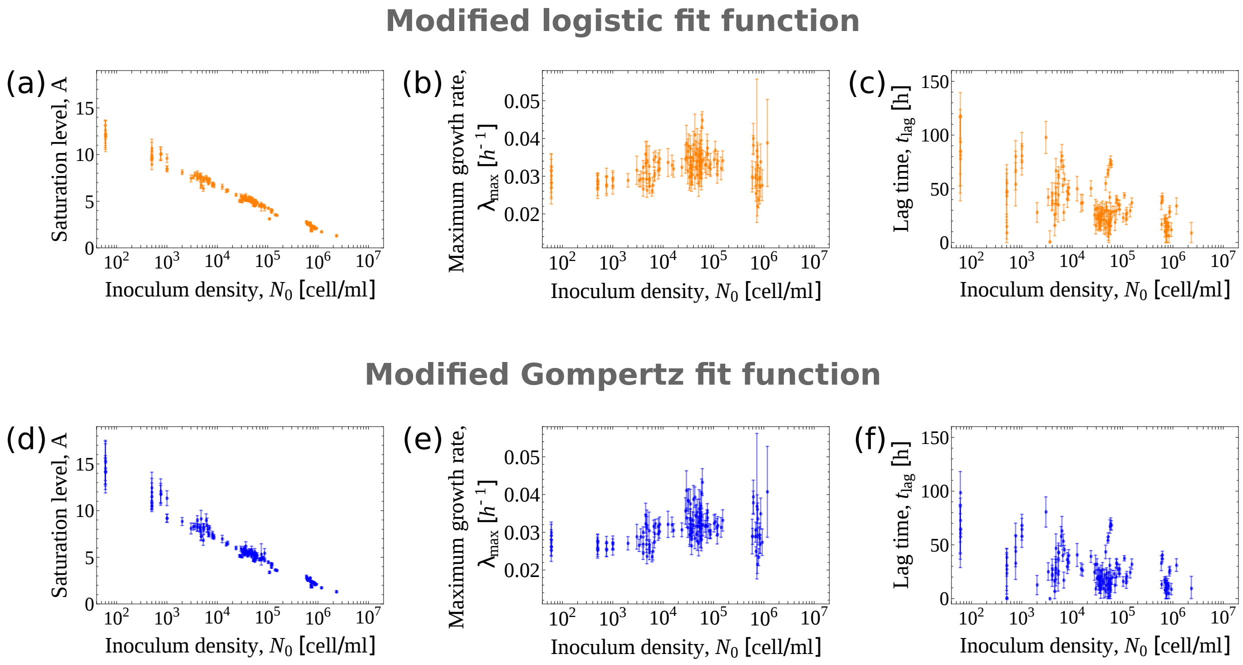

Figure S2. Growth parameters obtained by fitting experimental growth curves with the modified logistic function (a-c) and the modified Gompertz function (d-f). The saturation level A (a,d), maximum growth rate λ_{max} (b,e) and lag time t_{lag} (c,f) are plotted as a function of the inoculum density N_0. Each dot corresponds to a specific experiment and carries its own standard error.

Comparison between weighted and unweighted growth curve fit

All the growth curves that were fitted with a sigmoidal function contained from a minimum of 6 to a maximum of 22 data points. For all these curves we compared parameter estimates from unweighted fits (parameter estimates shown in the main text) to parameter estimates from weighted fits. Since each data point within the growth curves is the mean over many subsamples, the weights were measured as the inverse of the variance of the mean of the subsamples at each point (ex. variance of 15 subsample measurements divided by 15) on the log scale. As it is possible to notice from Figure S3, for most of the growth curves the parameter estimates from weighted and unweighted fits are indistinguishable (normal Z-test showed 95.5% of compatibility between the two estimates for all the parameters).

However, parameter estimates from growth curves with low initial density N_0 ($N_0 < 10^2$) looked much more spread for weighted fit than for unweighted fit. This is mainly due to the fact that for these curves a weighted fit does neglect too many points with the risk of doing overfitting. Figure S3a shows exactly this situation: the weighted fit (blue line) clearly disregards the first two points of the curve, thus fitting 5 points with a curve with 3 free parameters. In Figure S3b instead, weighted and unweighted fits look the same. While weighting data points with respect to their error bars makes sense when in presence of many data, in a situation in which all the data are informative, neglecting those that have bigger error bars may be misleading. For this reason we decided to leave in the main text the parameter estimates from unweighted fits.

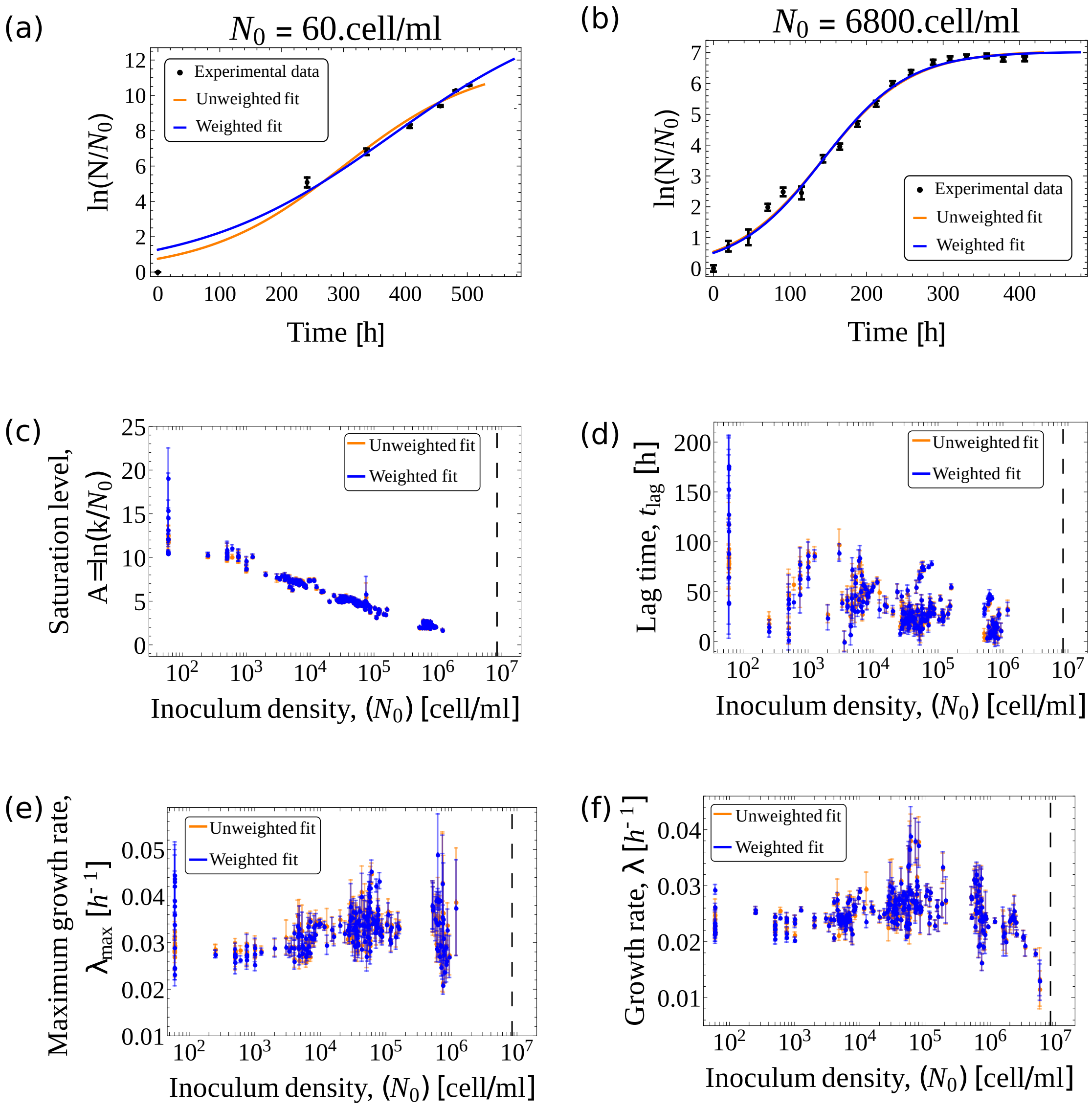

Figure S3. Comparison between weighted and unweighted fit of the growth curves. (a-b) Weighted (blue line) and unweighted (red line) fits for two different experiments. Black dots are experimental data together with their standard error of the mean. (c-f) Growth parameters are plotted against N_0. The parameters were obtained by performing weighted (blue dots) or unweighted (red dots) fits of the experimental growth curves with the modified logistic function. Each dot corresponds to a specific experiment and carries its own standard error.

Stirring experiments

Stirring experiments, in which clusters of cells were dissolved at regular intervals to test the role of mechanical interactions, were performed with Jurkat cells. Cells were seeded at initial density $N_0 \simeq 6 \cdot 10^4$ cells/ml, in the range of value of N_0 in which λ_{max} increases with the initial density. Three sets of experiments were performed by stirring populations at different frequencies: (i) three times per day (but counted only once); (ii) once every two days, and (iii) once every five days. In parallel to each of these conditions, a control experiment was run, where cells were stirred and counted once a day. The resulting growth curves are shown in Figure S4b-d, where data represent the mean over experimental replicates and error bars represent their dispersion. The superposition of each experiment (red dots) with its control (blue dots) suggests a negligible influence of clusters in the growth dynamics at the time scales considered.

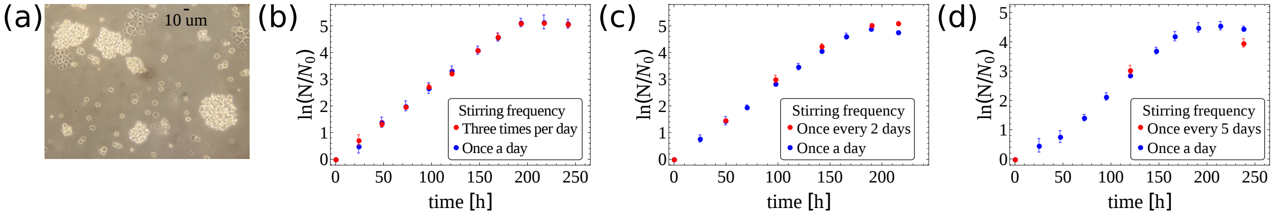

Figure S4. Results of stirring experiments. (a) Representative micrograph of Jurkat cell population growing in its standard growth medium. Clusters of cells are visible as well as isolated single cells. During stirring experiments clusters were dissolved at three different frequencies and counted every day. (b-d) Growth curves obtained from the experiments with stirring frequencies three times a day (b), once every two days (c), and once every five days (d), are shown as red markers. Blue markers represent the control, i.e. no stirring and counting every day. Each marker represents the average over three (b,d) or two (c) replicates, while the error bars denotes the data dispersion over the replicates.

Accuracy of the counting algorithm

Figure S5 shows an example of micrographs analysed during the experiments. We showed on the left an example of the raw phase contrast images for two different initial cell densities, while on the right the results of the counting algorithm, where red dots corresponded to the segmented (and counted) objects (cells).

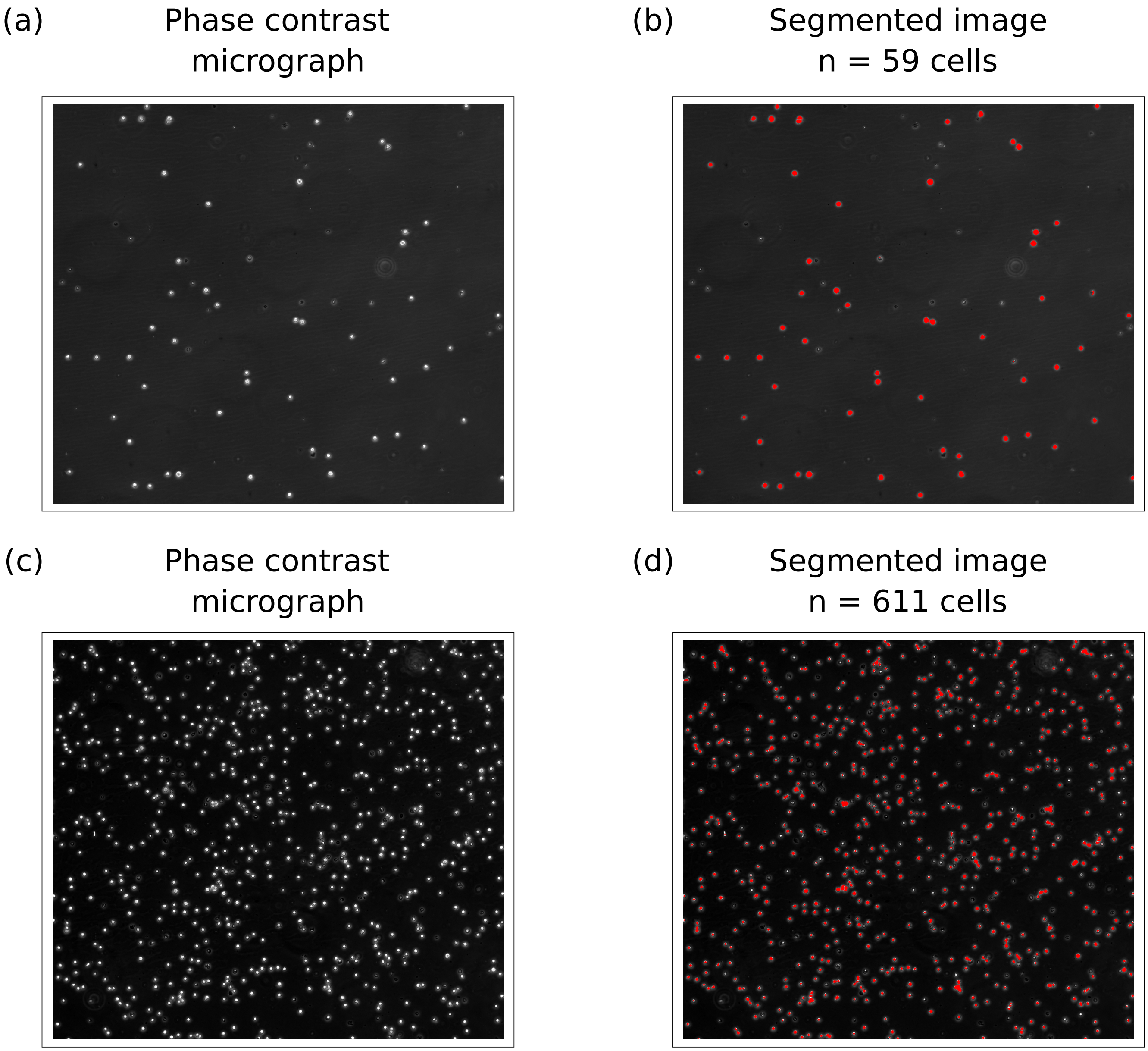

Figure S5. Micrographs analysed during the growth experiments. (a,c) Raw phase contrast images showing cells. (b,d) Results of the counting algorithm. Red dots correspond to segmented objects (cells).

We wondered if the algorithm designed to count cells within micrographs were unbiased with respect to cell density. To test the accuracy of the algorithm we then automatically generated and analysed images with a known amount of round-shaped objects with the same characteristics of the cells in terms of circularity and number of pixels ($>$ 10 pixels).

The algorithm used to generate images with round-shape objects was custom made and exploited MATLAB built-in functions. Starting from a black image with the same dimensions of the experimental micrographs, the algorithm required as input the radius ($r = 4$ pixels) and the number of objects (n) to generate, and then randomly assigned their coordinates before drawing them, see Figure S6a,c.

Figures S6b,d shows the result of the segmentation performed by the cell counting algorithm: red areas represent the

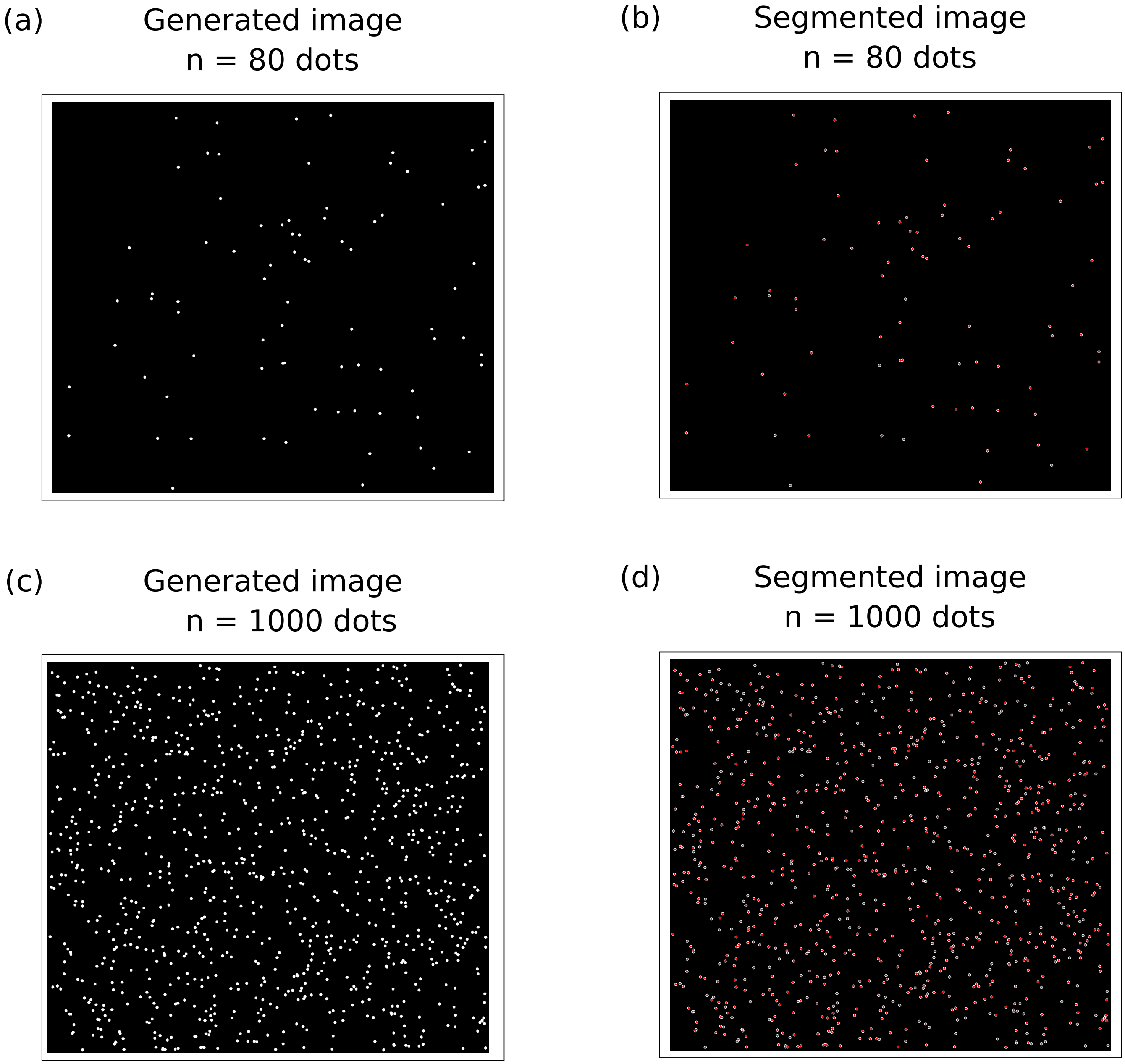

Figure S6. (a,c) Images with known amounts of round-shaped objects randomly distributed within the image surface used to test the cell counting algorithm. (b,d) Results of the counting algorithm. Red dots correspond to segmented objects.

recognized objects.

To test the accuracy, we then compared the known amount of objects (n) generated in the input images (15 images per each n) with the results of the automatic counting (n'). Figure S7a shows that for $n \leq 5000$, the values lied on the bisector (blue line in figure), suggesting good counting performance. However, such precision is lost when increasing the number of objects present within the image. This can also be visualized by looking at the CV (ratio between the standard deviation of the counted objects and their mean value): although very small, it increased when increasing n. This is mainly due to the fact that the overpopulation of space obtained by increasing n, led to a higher amount of touching objects (that cannot be resolved by the algorithm). Indeed, to experimentally overcome this issue, before every counting measurement we properly stirred and diluted the cells. Our accuracy test suggested that the algorithm we used to count cells was accurate within the range of cells considered experimentally (the higher amount of cells counted in our experiments was approximately 3000 cells per micrograph).

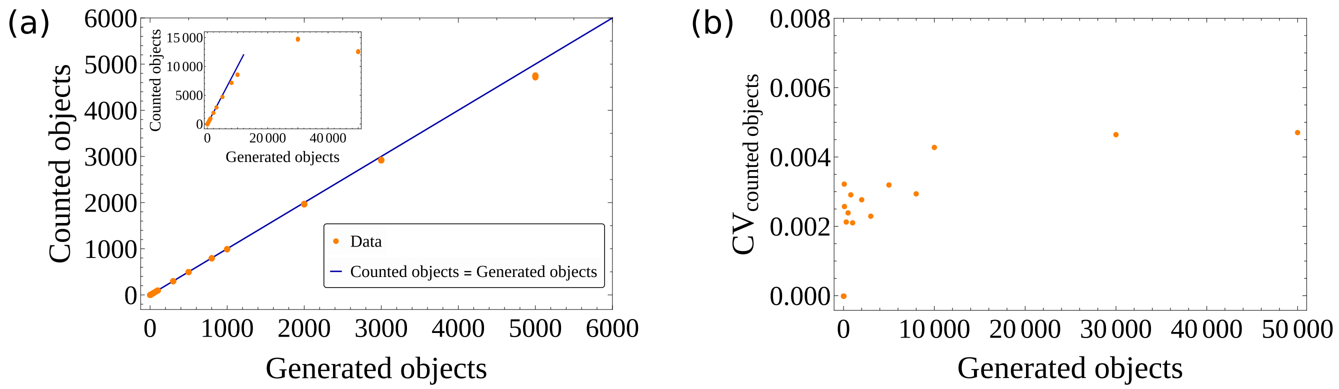

Figure S7. (a) Objects counted by the cell counting algorithm are shown against the known amount n of drawn objects. For $n > 5000$, data divert from the bisector. (b) CV of the counted objects evaluated over 15 different images as a function of the known amount n of generated objects.

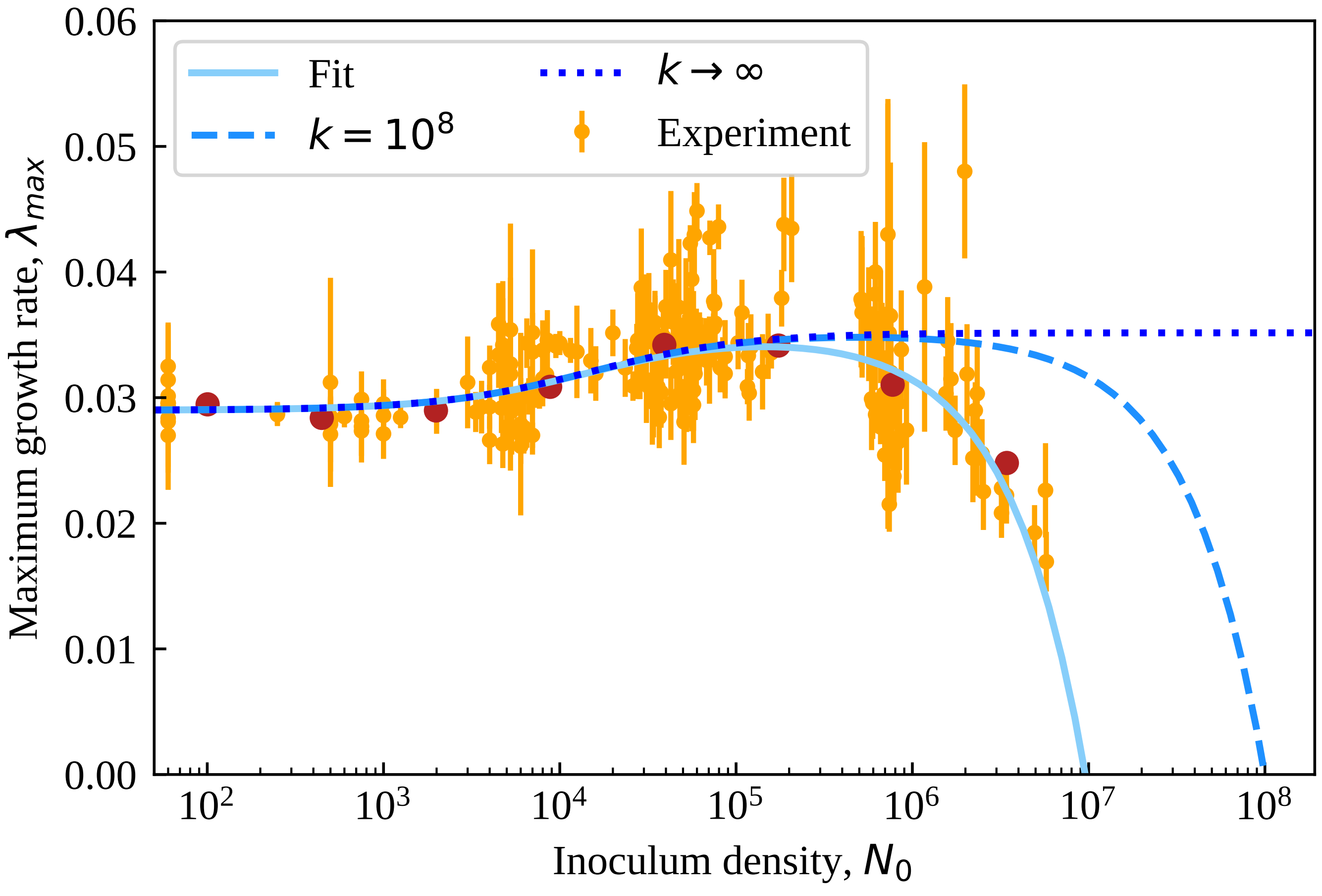

Figure S8. Behaviour of $\lambda_{\max}$ versus N_0 in individual experiments (orange markers), empirical means (red markers) and best weighted fit of experimental data to Eq. 3 of the Main Text (solid line) with $r_0 = 0.029$ and $\beta = 1$ (constrained), leaving δr, N_c and k as free parameters. Their optimal values are found to be $\delta r = (6.1 \pm 0.6) \times 10^{-3}$, $N_c = (1.5 \pm 0.5) \times 10^4$ and $k = (9.6 \pm 0.9) \times 10^6$. The dashed and dotted lines display the behaviour expected on the basis of Eq. 3 of the Main Text for the same value of all parameters except for k, which takes values of $k = 10^8$ (dashed line) and $k = \infty$ (dotted line), respectively. In essence, an increase of k alone will cause the growth rate to plateau as a function of N_0. If cooperation is strengthened, other parameters will also be modified. In such a case, one should expect a further increase of $\lambda_{\max}$ followed by a plateau.

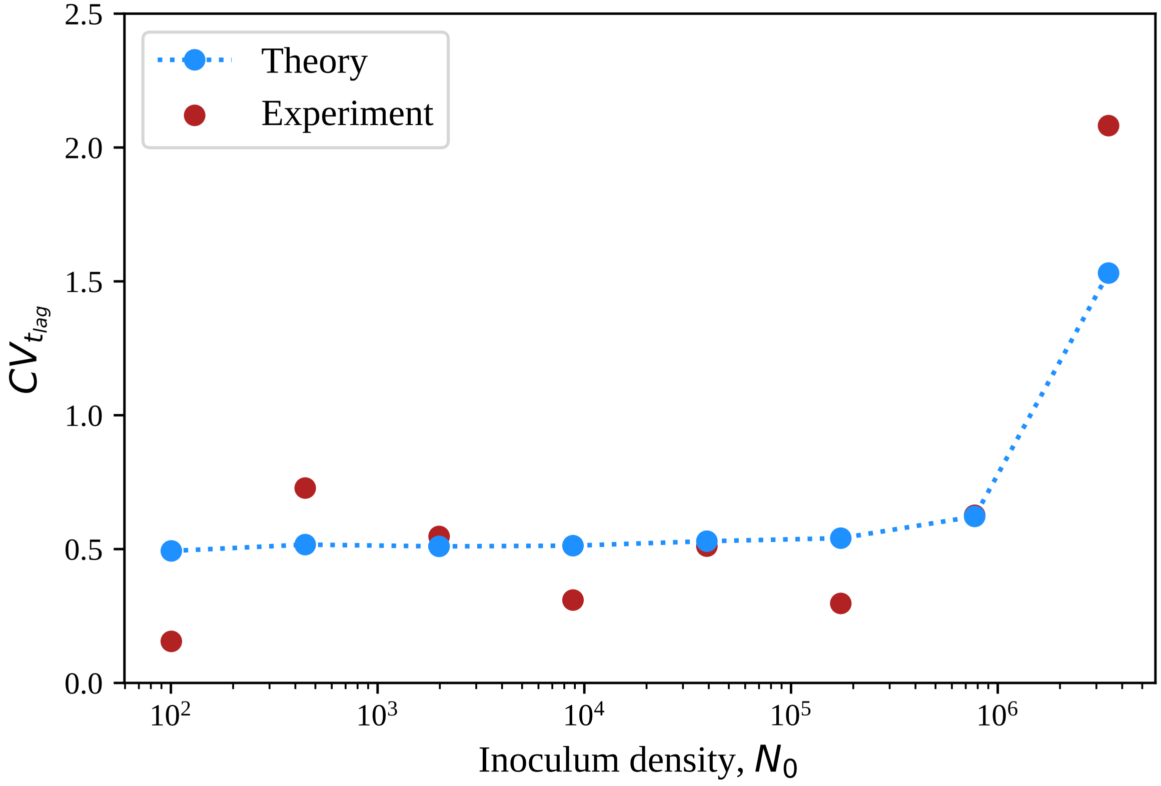

Figure S9. Empirical behaviour of $CV_{t_{\text{lag}}}$ versus N_0 (red markers) and best fit to Eq. (12) of the Main Text with $p = 0.38$. We used empirical values for $\lambda_{\text{max}} t_{\text{lag}}$ (varying across bins) and $CV_k = 0.44$, leaving $CV_{\lambda_{\text{max}}}$ as the only fitting parameter. Its optimal value, $(5 \pm 1) \times 10^{-1}$, is larger than the empirical relative fluctuations of λ_{max}. Notice however that in this way we are neglecting the fact that the coefficient of variation of λ_{max} is itself a function of N_0. This choice therefore yields a crude approximation for the true $CV_{t_{\text{lag}}}$.

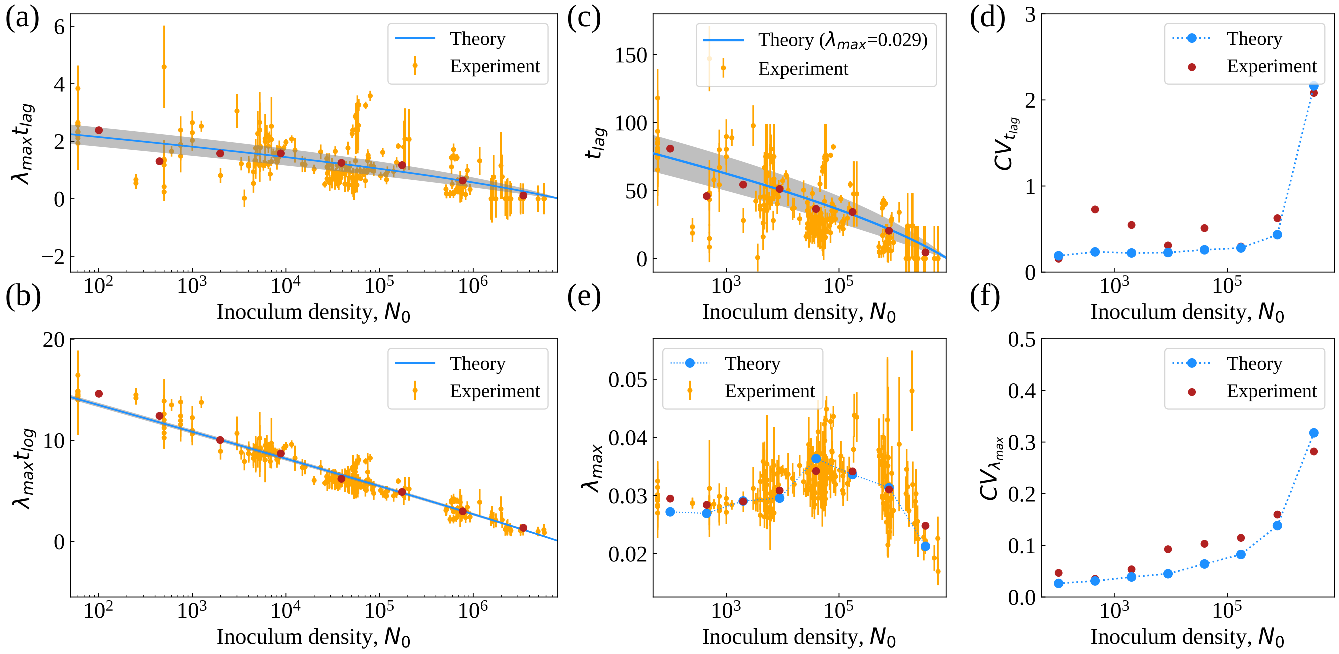

Figure S10. (a-b) Behaviour of $\lambda_{\max} t_{\text{lag}}$ (panel a) and $\lambda_{\max} t_{\log}$ (panel b) versus N_0 from experiments (orange markers), empirical means (red markers) and theoretical predictions based on Eq. 1 and 2 of the Main Text. For simplicity, we used for k the value $k = 8.4 \times 10^6$, corresponding to the empirical average carrying capacity. (c-d) Empirical behaviour of t_{lag} (panel c) and $\text{CV}_{t_{\text{lag}}}$ (panel d) versus N_0 (orange markers in (c)), empirical means (red markers) and best fit of experimental datapoints to Eq. 1 of the Main Text with $p = 0.38$. Grey shaded areas represent the 95% confidence intervals. For $\text{CV}_{t_{\text{lag}}}$ we used empirical values for $\lambda_{\max} t_{\text{lag}}$ (varying across bins) and $\text{CV}_{\lambda_{\max}} = 0.14$, leaving CV_k as the only fitting parameter. Its optimal value, $(9 \pm 1) \times 10^{-1}$, is of the same order of magnitude of the experimental one. (e-f) Empirical behaviour of $\lambda_{\max}$ (panel e) and $\text{CV}_{\lambda_{\max}}$ (panel f) versus N_0 (orange markers in (e)), empirical means (red markers) and best fit to Eq. 2 of the Main Text with $p = 0.38$ and $t_{\log}$ set to its empirical value in each bin. For $\text{CV}_{\lambda_{\max}}$ we used empirical values for $\lambda_{\max} t_{\log}$ (varying across bins) and $\text{CV}_{t_{log}} = 0$, leaving CV_k as the only fitting parameter. Its optimal value, $(3.4 \pm 0.3) \times 10^{-1}$ is of the same order of magnitude as the experimental one.

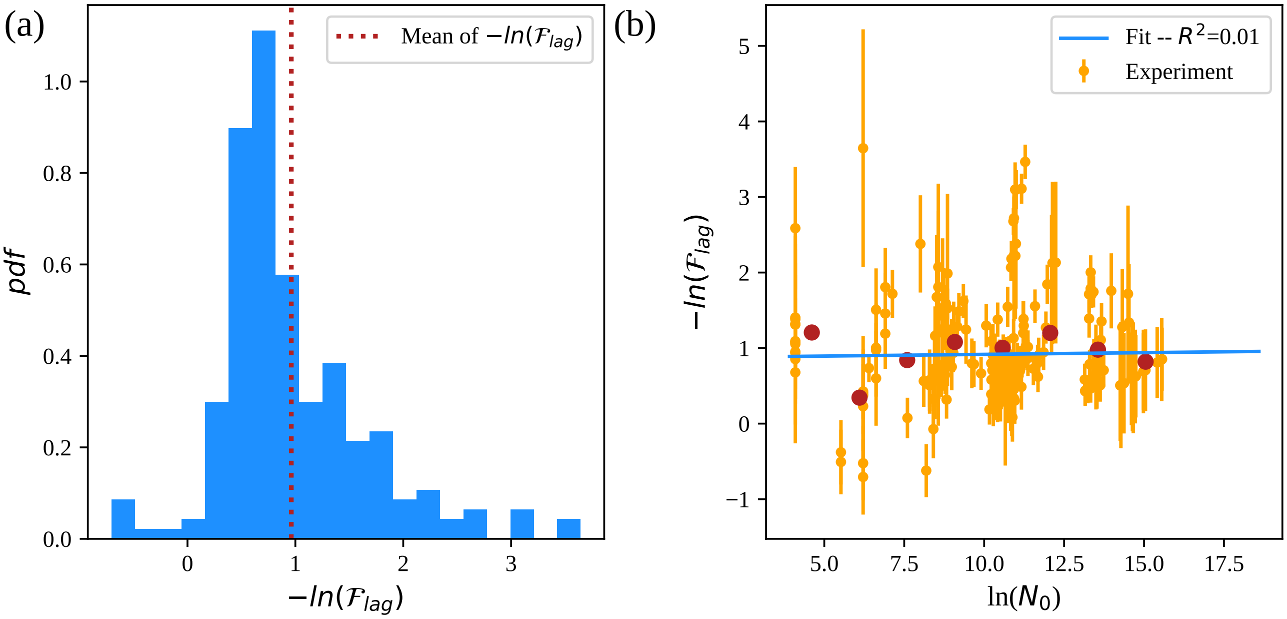

Figure S11. (a) Distribution of $-\ln(\mathscr{F}_{\text{lag}})$ computed from experimental data using Eq. (9) of the Main Text with $p = 0.38$. The red dotted line marks the empirical mean of the distribution. (b) Experimental data (orange markers), empirical means (red markers) and best linear weighted fit of experimental datapoints (blue line) of $-\ln(\mathscr{F}_{\text{lag}})$ as a function of the logarithm of the inoculum density, N_0. The value of R^2 suggests that the approximation $-\ln(\mathscr{F}_{\text{lag}}) \simeq 1$ employed in our analysis is very accurate.

References

1. Zwietering, M. H., Jongenburger, I., Rombouts, F. M. & van 't Riet, K. Modeling of the bacterial growth curve. *Appl. Environ. Microbiol.* **56**, 1875–1881 (1990). URL http://www.ncbi.nlm.nih.gov/pmc/articles/PMC184525/.
